# Drift of Thought

**Published:** 1897-01

**Authors:** 


### DRIFT OF THOUGHT.

THE popularity of cycling and the good effects on the health of its devotees from exercise in the open air is now awakening a keener interest in horseback riding than ever before. All the popular Eastern seaside resorts the past summer gave employment to an unusually large number of riding-masters and instructors, and the riding-schools in the Eastern cities are full to overflowing. Its good influence in 1897 will prove a boon to the horse-market and likewise to the carriage and fancy trap builders.

Another veterinarian proposes to enter the field of proprietary medicine in the early spring, and, not to be outdone by any of his competitors, will send out a coupon with each package of his nostrums, which, for a two-cent stamp, will insure the purchaser of each package a free consultation; and, as this will sell another of the same make, it promises to outrank the wager-plan or "money-refunded" scheme. Wait for the reaction.

The *Breeders' Gazette* of December 23d makes a strong appeal for the establishment of a complete veterinary college at the State Agricultural College at Champaign, Ill., and believes that the time is opportune, the need great, and urges action upon all those interested. It demands a school of the highest character and complete in every department of veterinary science. We are strongly inclined to look for the best results and the greatest permanency of veterinary institutions as departments of State universities or agricultural colleges.

The canton of Zurich has decided to prohibit the importation of American canned meats by adopting a regulation forbidding the use of borax as a meat-preservative. Some remote scientific cause must be found for cutting off this valuable trade of the live-stock growers of our country, while exporting to us beet-roots, adulterated wines, our own cotton-seed oil as olive oil, etc. When will our people learn how to deal with these injured innocents who cunningly drain away our millions?

